# SF3B4 stabilizes SREBF1 via 3′UTR binding to drive hepatocellular carcinoma progression

**DOI:** 10.3389/fonc.2026.1833357

**Published:** 2026-05-21

**Authors:** Yuan Fang, Dan Wang, Lei Han, Mengge Li, Qiuyue He, WangGan Xu, HaiJing Li, Zhong Zeng, Jie Lin, HanFei Huang

**Affiliations:** 1 Organ Transplantation Center, the First Affiliated Hospital of Kunming Medical University, Kunming, Yunnan, China; 2 State Key Laboratory of Systems Medicine for Cancer, Shanghai Cancer Institute, Renji Hospital, Shanghai Jiao Tong University School of Medicine, Shanghai, China; 3 Department of Clinical Laboratory, The First Affiliated Hospital of Kunming Medical University, Kunming, China

**Keywords:** alternative splicing events, hepatocellular carcinoma, metabolic reprogramming, SF3B4, SREBF1

## Abstract

**Background:**

Hepatocellular carcinoma (HCC) represents a leading cause of cancer-associated deaths worldwide, and its development is strongly associated with aberrant RNA processing and metabolic reprogramming. Splicing factor 3B subunit 4 (SF3B4), the core component of the U2 spliceosome, has been implicated in tumorigenesis; however, its post-transcriptional functions and role in metabolic regulation in HCC remain unclear.

**Methods:**

SF3B4 expression and its prognostic significance were assessed using TCGA pan-cancer datasets and clinical HCC samples. SF3B4 was either silenced or overexpressed in HCC cell lines, followed by full-length transcriptome sequencing via Oxford Nanopore Technology to analyze global transcriptional and alternative splicing changes. SF3B4–RNA interactions were examined using RNA immunoprecipitation, dual-luciferase reporter assays, RNA pull-down, and mRNA stability assays. Functional assays assessed cell proliferation, apoptosis, invasion, and migration. Rescue experiments involved overexpressing SREBF1 in SF3B4-silenced cells. *In vivo* tumorigenic effects were validated using xenograft mouse models.

**Results:**

SF3B4 expression was significantly elevated in HCC tissues and correlated with poor OS and DFS. Transcriptomic analyses showed that SF3B4 knockdown induced widespread transcriptional remodeling and extensive alternative splicing reprogramming. Integrative analyses identified sterol regulatory element-binding transcription factor 1 (SREBF1) as a direct downstream target of SF3B4. Mechanistically, SF3B4 bound directly to the 3′ untranslated region (3′UTR) of SREBF1 mRNA, enhancing its stability and expression. Functional assays demonstrated that SREBF1 promoted HCC cell proliferation, invasion, and migration, while inhibiting apoptosis. Notably, SREBF1 overexpression partially rescued the malignant phenotypes and transcriptomic alterations induced by SF3B4 knockdown. *In vivo*, SF3B4 silencing significantly inhibited tumor growth and reduced SREBF1 expression in xenograft models. Clinical validation confirmed the co-upregulation of SF3B4 and SREBF1 in HCC tissues.

**Conclusion:**

This study reveals a novel post-transcriptional mechanism in which SF3B4 promotes HCC progression by stabilizing SREBF1 mRNA through direct 3′UTR binding. The SF3B4-SREBF1 axis connects RNA metabolism dysregulation to lipid metabolic reprogramming, offering new mechanistic insights and potential therapeutic targets for HCC.

## 1 Introduction

Hepatocellular carcinoma (HCC) is a major contributor to global cancer-related incidence and mortality, especially in areas with a high burden of viral hepatitis (1). Despite advancements in monitoring, surgical techniques, molecular targeted therapies, and immunotherapy, the prognosis for HCC remains poor due to late diagnosis, rapid progression, and high recurrence rates (2). Accordingly, elucidating the molecular basis of HCC development and progression is imperative to refine diagnostic methods, optimize treatment strategies, and improve patient prognosis (3).

Splicing factors are central regulators of gene expression, particularly in pre-mRNA splicing, where they facilitate intron removal and exon joining, influencing transcript maturation and diversity (4). In tumor cells, splicing patterns are often reprogrammed, leading to the production of dysfunctional splice variants that drive tumorigenesis, progression, drug resistance, and immune escape (5). Among RNA-binding proteins (RBPs), splicing factors are pivotal in controlling exon-intron recognition, spliceosome assembly, and transcriptomic diversity (6). Dysregulated splicing factor expression in tumor cells can lead to selective splicing errors. For example, proteins such as SRSF1, members of the hnRNP family, and RBM proteins are closely associated with tumor cell proliferation, epithelial-mesenchymal transition (EMT), and metabolic reprogramming (7). PCBP1 regulates the transcription and alternative splicing (AS) of genes linked to liver cancer metastasis (8). Additionally, the RBP RALY activates the cholesterol synthesis pathway *via* MTA1 splicing in HCC and may represent a promising therapeutic target (9). In addition, reduced levels of pro-inflammatory cytokines, particularly IL-6, are associated with improved recovery and prognosis, whereas elevated IL-6 correlates with tumor recurrence and unfavorable disease-free survival, highlighting the clinical relevance of inflammatory and metabolic regulation in HCC (10).

Splicing factor 3B subunit 4 (SF3B4), a core component of the SF3b complex in the U2-type spliceosome, plays a pivotal role in early splice site recognition (11). It not only contributes to normal splicing but may also influence post-transcriptional stability (12), nuclear export (13), and translation through RNA binding, thereby impacting the biological behavior of tumor cells (14). Increasing research has focused on SF3B4 in various cancers, including lung adenocarcinoma (15) and ovarian cancer (8), where its aberrant overexpression promotes oncogenic phenotypes (16). Our previous study demonstrated that SF3B4 regulates AS and directly interacts with oncogenic transcripts such as TRIM28 and SETD5, influencing proliferation, apoptosis, and RNA processing pathways (17). However, the broader post-transcriptional functions of SF3B4 and its potential interaction with metabolic regulators in HCC remain largely unexplored.

Current research suggests that AS holds promise as a novel antigen source for patients with liver cancer (18). The exploration of SF3B4 offers new avenues for developing mRNA vaccines and advancing early diagnosis and treatment strategies for liver cancer, highlighting its significance in achieving effective immunotherapy.

In this study, we aimed to elucidate the functional role and underlying mechanisms of SF3B4 in hepatocellular carcinoma. Using integrative transcriptomic analyses combined with molecular and cellular assays, we demonstrate that SF3B4 promotes HCC progression by directly binding to the 3′UTR of SREBF1 mRNA and enhancing its stability. This interaction leads to increased SREBF1 expression and subsequent metabolic reprogramming, thereby facilitating tumor growth and metastasis. Furthermore, rescue experiments confirm that SREBF1 is a critical downstream effector of SF3B4. Collectively, our findings reveal a novel post-transcriptional regulatory mechanism linking RNA processing factors to metabolic regulation in HCC and highlight the SF3B4–SREBF1 axis as a potential therapeutic target.

## 2 Methods

### 2.1 Cell culture and transient transfection

Huh-7 cells (Procell, China) were cultured with 10% fetal bovine serum (FBS; Gibco, Thermo Fisher Scientific, USA) and 1% penicillin–streptomycin (Gibco, Thermo Fisher Scientific, USA) at 37 C in a humidified incubator with 5% CO_2_. Only exponentially growing cells were used for all assays. SiRNAs (GenePharma, Shanghai, China) targeting SF3B4 or SREBF1 and a negative control siRNA were synthesized commercially (**Table 1**). The complete coding sequence of human SREBF1 (GenePharma, Shanghai, China) was cloned into the pcDNA3.1 expression vector. Lipofectamine™ 2000 (Invitrogen, CA, USA) was used for transient transfection as recommended by the manufacturer. Cells were harvested 48h later for downstream analyses.

**Table 1:** The sequences of siRNAs used for gene silencing.

	Sense	Anti-sense
siNC	UUCUCCGAACGUG UCACGUTT	ACGUGACACGUUCG GAGAATT
siSF3B4	GCACCAAGGCUAUG GCUUUTT	AAAGCCAUAGCCUUG GUGCTT
siSREBF1-1	CUGAGGCAAAGCUG AAUAATT	UUAUUCAGCUUUGC CUCAGTT
siSREBF1-2	GCUGCAUUGAGAGU GAAGATT	UCUUCACUCUCAAUG CAGCTT

### 2.2 RNA extraction and quantitative real-time PCR

RNA was purified using TRIzol reagent following standard protocols. Concentration and purity were measured spectrophotometrically, and integrity was confirmed by gel electrophoresis. cDNA synthesis was performed under sequential incubation at 42 C (5min), 37 C (15min), and 85 C (5 s). qRT–PCR was conducted on an ABI QuantStudio™ 5 system using SYBR Green chemistry (**Table 2**). The amplification protocol consisted of an initial denaturation at 95 C for 10 minutes, followed by 40 cycles of 95 C for 15 seconds and 60 C for 1 minute. Each sample was analyzed in triplicate. Relative gene expression levels were calculated using the 2^−ΔΔCt^ method, with GAPDH as the internal control.

**Table 2:** The primers used for quantitative RT-PCR.

Name	Sequence (5’-3’)
GAPDH	TCAAGAAGGTGGTGAAGCAGG
	TCAAAGGTGGAGGAGTGGGT
SF3B4	TCAACACCCACATGCCAAAG
SREBF1	CTGATGCTTTGTTCACCCGT GCCCCTGTAACGACCACTG CAGCGAGTCTGCCTTGATG

### 2.3 mRNA stability assay

For analysis of mRNA stability, transcription was inhibited by treating cells with actinomycin D. Total RNA was collected at specified time points after treatment, and SREBF1 mRNA levels were quantified by qRT–PCR. Transcript levels were normalized to the levels at time zero, and mRNA decay curves were plotted using GraphPad Prism software.

### 2.4 Western blot analysis

Proteins were isolated using lysis buffer containing protease inhibitors. Following quantification, equal protein amounts were resolved by SDS–PAGE and transferred onto PVDF membranes. Membranes were blocked in 5% non-fat milk and incubated at 4 C overnight with primary antibodies against SF3B4, β-actin, and GAPDH at the indicated dilutions. After incubation with HRP-linked secondary antibodies, protein bands were visualized using an ECL detection system.

### 2.5 Dual-luciferase reporter assay

Wild-type and mutant SREBF1 3′UTR sequences were cloned into luciferase reporter vectors. Huh-7 cells were co-transfected with these constructs together with SF3B4 expression or control plasmids. After 48 h, luciferase signals were quantified using a dual-luciferase system, with firefly luciferase values normalized against Renilla luciferase.

### 2.6 RNA immunoprecipitation and RNA pull-down assays

RIP assays were performed using antibodies against SF3B4 to examine RNA–protein interactions. Co-precipitated RNAs were extracted and analyzed by qRT–PCR. For RNA pull-down assays, biotin-labeled sense or antisense probes targeting SREBF1 mRNA were incubated with cell lysates, followed by streptavidin bead capture and Western blot detection of SF3B4.

### 2.7 Cell proliferation, apoptosis, migration, and invasion assays

Proliferative capacity was examined using an EdU assay. Apoptotic cells were detected by Annexin V/PI staining and quantified via flow cytometry. Transwell chambers were employed to assess migration and invasion, with Matrigel pre-coating applied for invasion assays. Cells passing through the membrane were fixed, stained, and quantified under a microscope.

### 2.8 *In vivo* xenograft tumor model

Male mice (Shanghai SLAC Laboratory Animal Co. Ltd.) were housed under specific pathogen-free conditions. Huh-7 cells transfected with control or si-SF3B4 were suspended in serum-free medium mixed with Matrigel and subcutaneously injected into the axillary region of the mice (5 × 10^6^ cells per mouse). Tumor volume was measured every two days. At the study endpoint, tumors were excised for further analysis.

### 2.9 Immunohistochemistry

Tumor tissues were fixed, embedded in paraffin, and sectioned. After deparaffinization and antigen retrieval, sections were incubated with primary antibodies against SF3B4 or SREBF1, followed by HRP-conjugated secondary antibodies. Signals were visualized using DAB substrate and counterstained with hematoxylin.

### 2.10 Statistical analysis

Data were analyzed using GraphPad Prism 8.0 and are shown as mean ± SD. Intergroup differences were evaluated by one-way ANOVA with *post hoc* testing. P < 0.05 was considered significant.

## 3 Results

### 3.1 The expression level of SF3B4 in pan-cancer

**Figure 1:**
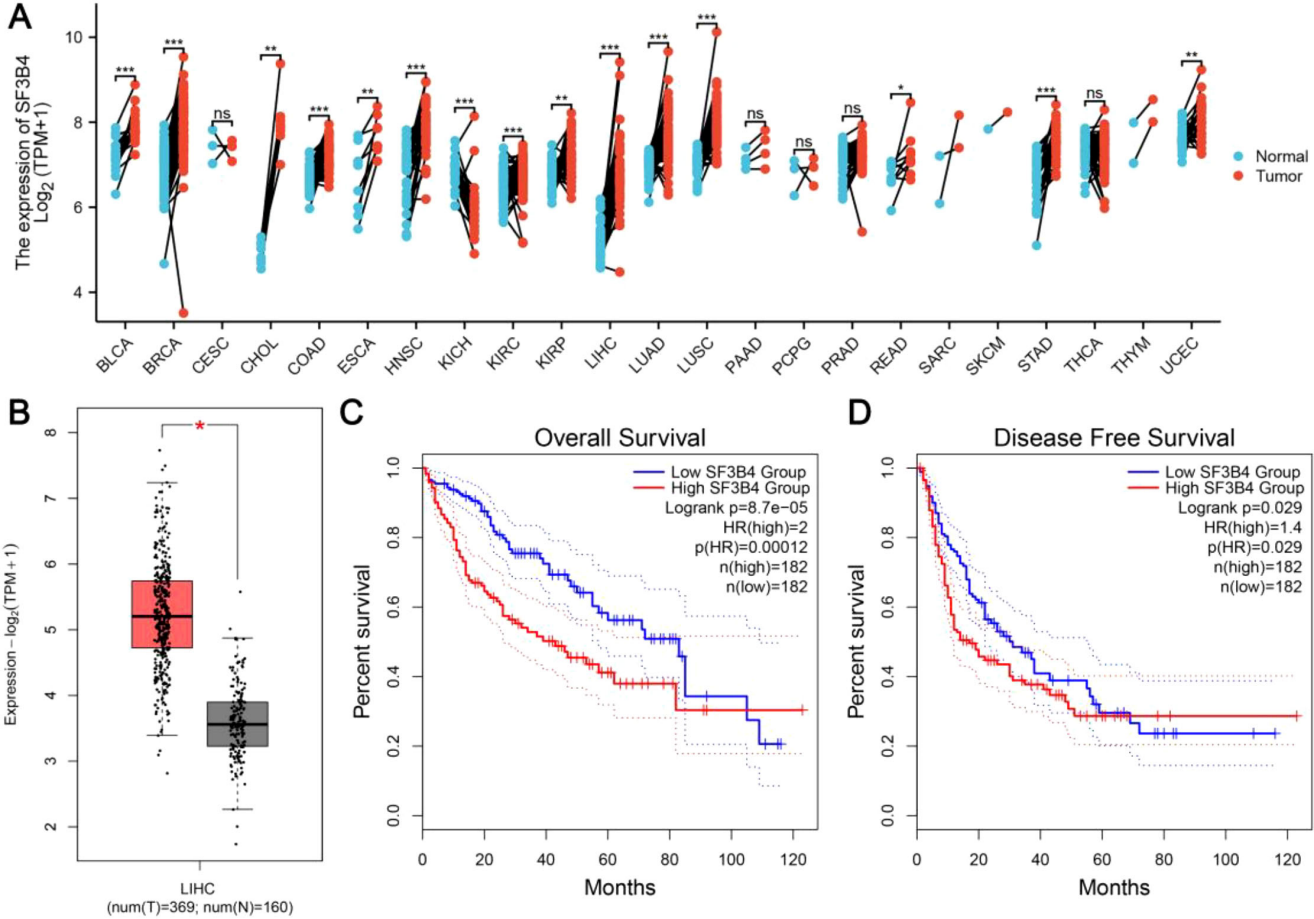
SF3B4 is upregulated in HCC and associated with poor prognosis. **(A)** Pan-cancer analysis of SF3B4 expression in tumor and normal tissues from TCGA datasets. **(B)** SF3B4 expression in LIHC tumor (T) and normal (N) tissues. **(C)** Kaplan–Meier analysis of OS stratified by SF3B4 expression in LIHC. **(D)** Kaplan–Meier analysis of DFS according to SF3B4 expression.

Pan-cancer analysis of TCGA datasets revealed that SF3B4 expression was significantly upregulated across various tumor types compared to corresponding normal tissues (**Figure 1A**). Notably, SF3B4 expression was markedly higher in liver hepatocellular carcinoma (LIHC). Consistent with this pan-cancer analysis, SF3B4 expression was significantly elevated in LIHC tumor tissues compared to normal liver tissues (**Figure 1B**). Survival analysis further indicated that patients with high SF3B4 expression had significantly poorer overall survival (OS) compared to those with low SF3B4 expression (log-rank p = 8.7×10^–5^; HR = 2.0; **Figure 1C**). Additionally, high SF3B4 expression was associated with shorter disease-free survival (DFS) (log-rank p = 0.029; HR = 1.4; **Figure 1D**).

### 3.2 The differentially expressed genes in the full-length transcriptome of si-SF3B4 in HCC

**Figure 2:**
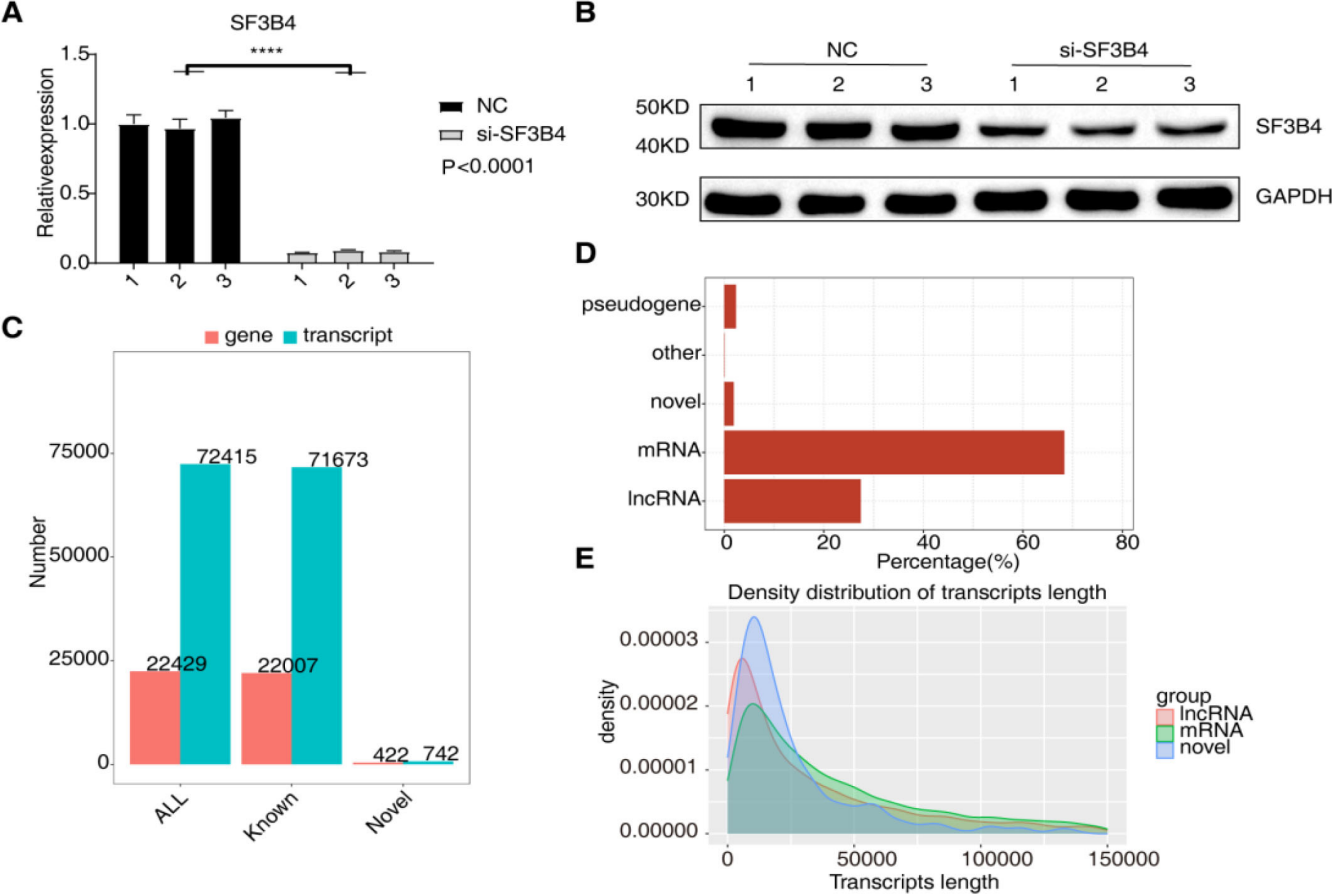
Validation of SF3B4 knockdown and transcriptome profiling in Huh-7 cells. **(A)** qRT–PCR analysis confirming the knockdown efficiency of SF3B4 in Huh-7 cells. **(B)** Western blot analysis verifying SF3B4 protein downregulation in Huh-7 cells after si-SF3B4 transfection. **(C)** Numbers of detected genes and transcripts identified by full-length transcriptome sequencing. **(D)** Proportional distribution of detected gene types, including mRNA, lncRNA, novel transcripts, pseudogenes, and others. **(E)** Length distribution of lncRNA, mRNA, and novel transcripts.

SF3B4 knockdown in Huh-7 cells was achieved using siRNA, and mRNA expression levels of SF3B4 were assessed by RT-qPCR. The results showed that, compared to the negative control (NC), si-SF3B4 treatment significantly reduced SF3B4 mRNA expression (*P* < 0.0001) (**Figure 2A**). Western blot analysis confirmed that SF3B4 protein expression was also significantly reduced in the si-SF3B4 group, demonstrating effective knockdown (**Figure 2B**). To ensure the reliability of subsequent transcriptome sequencing, quality control and statistical analyses were conducted on the raw data. The gene and transcript distribution (**Figure 2C**) revealed the detection of approximately 72,415 known genes and 71,673 known transcripts. Additionally, 422 new genes and 742 new transcripts were identified, indicating high data coverage. The distribution of transcript categories (**Figure 2D**) showed that mRNA comprised the largest proportion (over 70%), followed by lncRNA and other categories, consistent with the known transcriptional characteristics of Huh-7 cells. Transcript length distribution (**Figure 2E**) exhibited a right-skewed pattern for mRNA, lncRNA, and novel transcripts, with mRNA being longer and lncRNA being relatively shorter, consistent with typical high-throughput sequencing data characteristics.

### 3.3 Full-length transcriptome sequencing revealed the expression of the SF3B4 and transcripts in HCC

**Figure 3:**
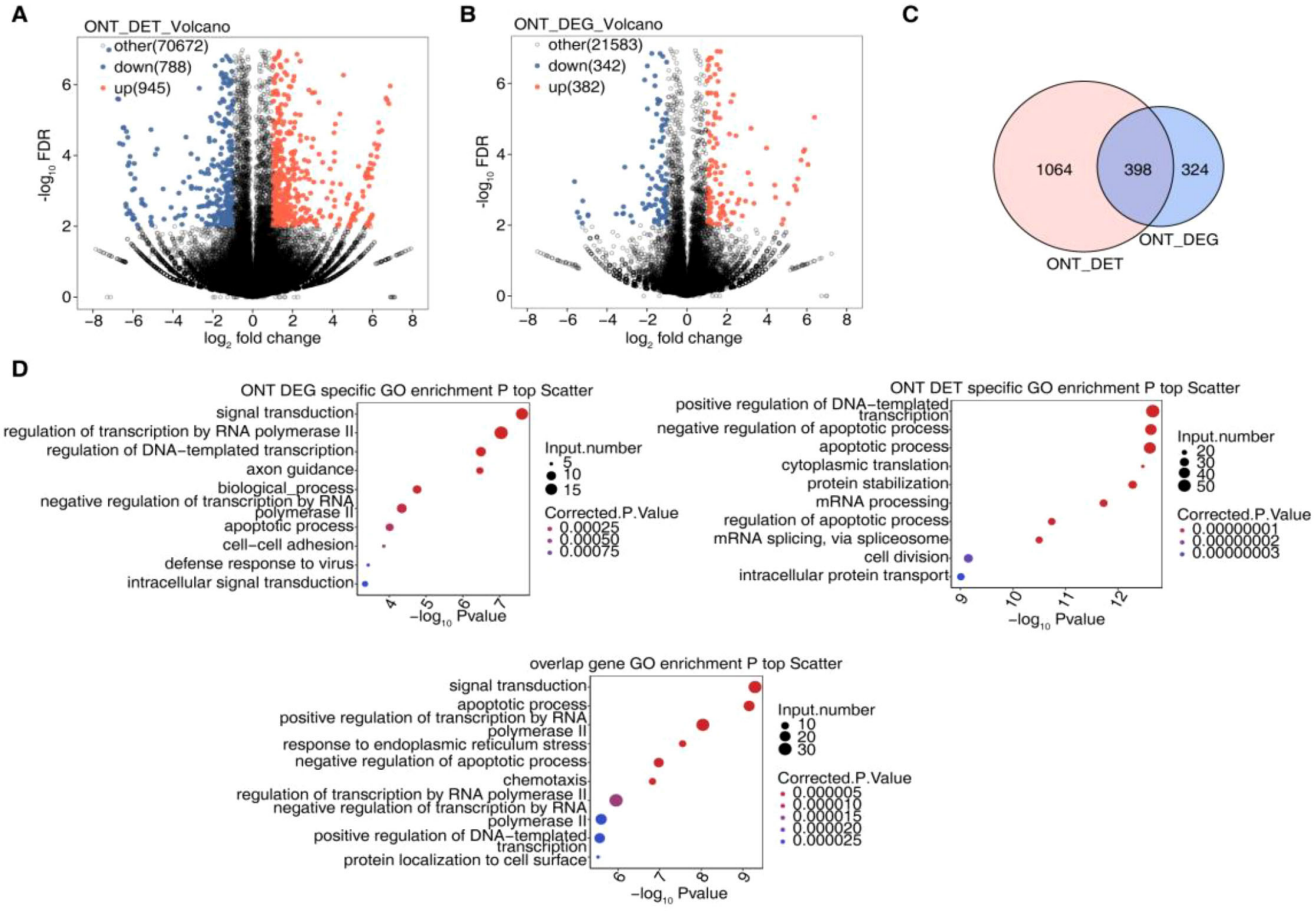
Differentially expressed genes and transcripts identified by ONT long-read RNA sequencing after SF3B4 knockdown. **(A)** Volcano plot showing DETs identified by ONT long-read RNA sequencing in si-SF3B4–treated cells compared with controls. **(B)** Volcano plot showing DEGs following SF3B4 knockdown. **(C)** Venn diagram illustrating the overlap between differentially expressed transcripts (DETs) and DEGs. **(D)** GO biological process enrichment analysis of DEGs, including DEG-specific, DET-specific, and overlapping gene sets.

Following SF3B4 knockdown in Huh-7 cells, transcriptome analysis was conducted using ONT long-read RNA sequencing technology. The results revealed significant transcriptional reprogramming upon SF3B4 silencing. Volcano plot analysis (**Figures 3A, B**) showed detection of approximately 72,000 transcripts, with 945 significantly upregulated and 788 significantly downregulated (|log2FC| > 1, FDR < 0.05). At the gene level, 21,500 genes were detected, of which 382 were significantly upregulated and 342 were significantly downregulated (|log2FC| > 1, FDR < 0.05), indicating that SF3B4 broadly regulates both transcript and gene expression. Intersection analysis of differentially expressed transcripts (DET) and differentially expressed genes (DEGs) (**Figure 3C**) revealed 398 genes with significant differential expression at both the transcript and gene levels, suggesting these may be direct regulatory targets of SF3B4. To explore the potential functions of SF3B4 in HCC, Gene Ontology (GO) enrichment analysis was performed on DETs, DEGs, and their intersection genes (**Figure 3D**). The analysis revealed that DEGs were predominantly enriched in pathways related to signal transduction, DNA-dependent transcriptional regulation, cell adhesion, axon guidance, and apoptosis. DETs were primarily enriched in mRNA splicing, cytoplasmic translation, cell division, DNA template transcriptional regulation, and apoptosis. Intersection genes were mainly involved in signal transduction, RNA polymerase II transcriptional regulation, endoplasmic reticulum stress, and apoptosis.

### 3.4 SF3B4 binds to regulate the expression of target genes in HCC

**Figure 4:**
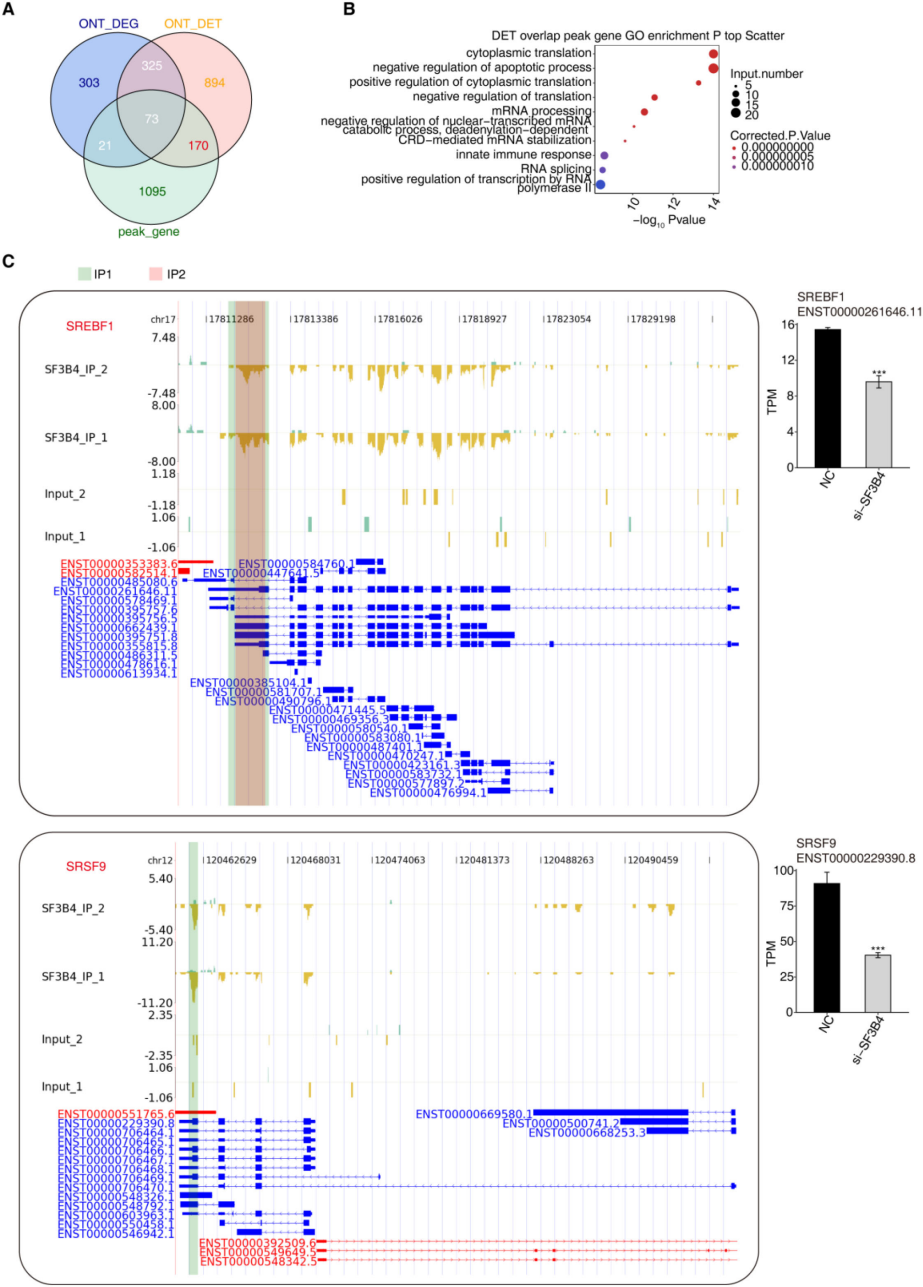
Identification of SF3B4-bound and SF3B4-regulated target genes. **(A)** Venn diagram showing the overlap among DEGs and DETs identified by ONT long-read RNA sequencing and peak-associated genes identified by iRIP-seq. **(B)** GO biological process enrichment analysis of genes overlapping between ONT RNA-seq DETs and iRIP-seq peak-associated genes. **(C)** Representative examples of SF3B4 target genes, SREBF1 and SRSF9, showing differential transcript expression (bar plots) and corresponding iRIP-seq peak read distributions. ***p<0.001.

Following SF3B4 knockdown in Huh-7, a combined analysis of ONT long-read RNA sequencing and iRIP-Seq data was performed to investigate DEGs, DETs, and SF3B4 binding peaks. The results demonstrated that SF3B4 plays a significant role in transcriptional regulation (**Figures 4A-C**). Initially, an intersection analysis was conducted between DEGs, DETs, and SF3B4 binding peak genes (**Figure 4A**). This analysis revealed that 73 genes were present in all three datasets, suggesting these genes may be direct targets of SF3B4 regulation. GO functional enrichment analysis of the overlapping DET and peak genes (**Figure 4B**) showed significant enrichment in pathways related to cytoplasmic translation, mRNA processing, RNA splicing, transcriptional regulation, apoptosis, and immune response. This suggests that SF3B4 may directly bind and regulate transcript processing and translation, contributing to various liver cancer-related biological functions. Furthermore, by combining SF3B4 binding peak data with transcriptome expression profiles, SREBF1 and SRSF9 were identified as potential direct downstream targets of SF3B4 (**Figure 4C**). In regions of SF3B4 binding sites, SF3B4 knockdown significantly reduced both its binding signal and the transcriptional levels of these two genes (*P* < 0.001), indicating that SF3B4 regulates their expression through direct binding.

### 3.5 Presentation of differential alternative splicing results of si-SF3B4 vs NC-ONt-lRRNA-SEq

**Figure 5:**
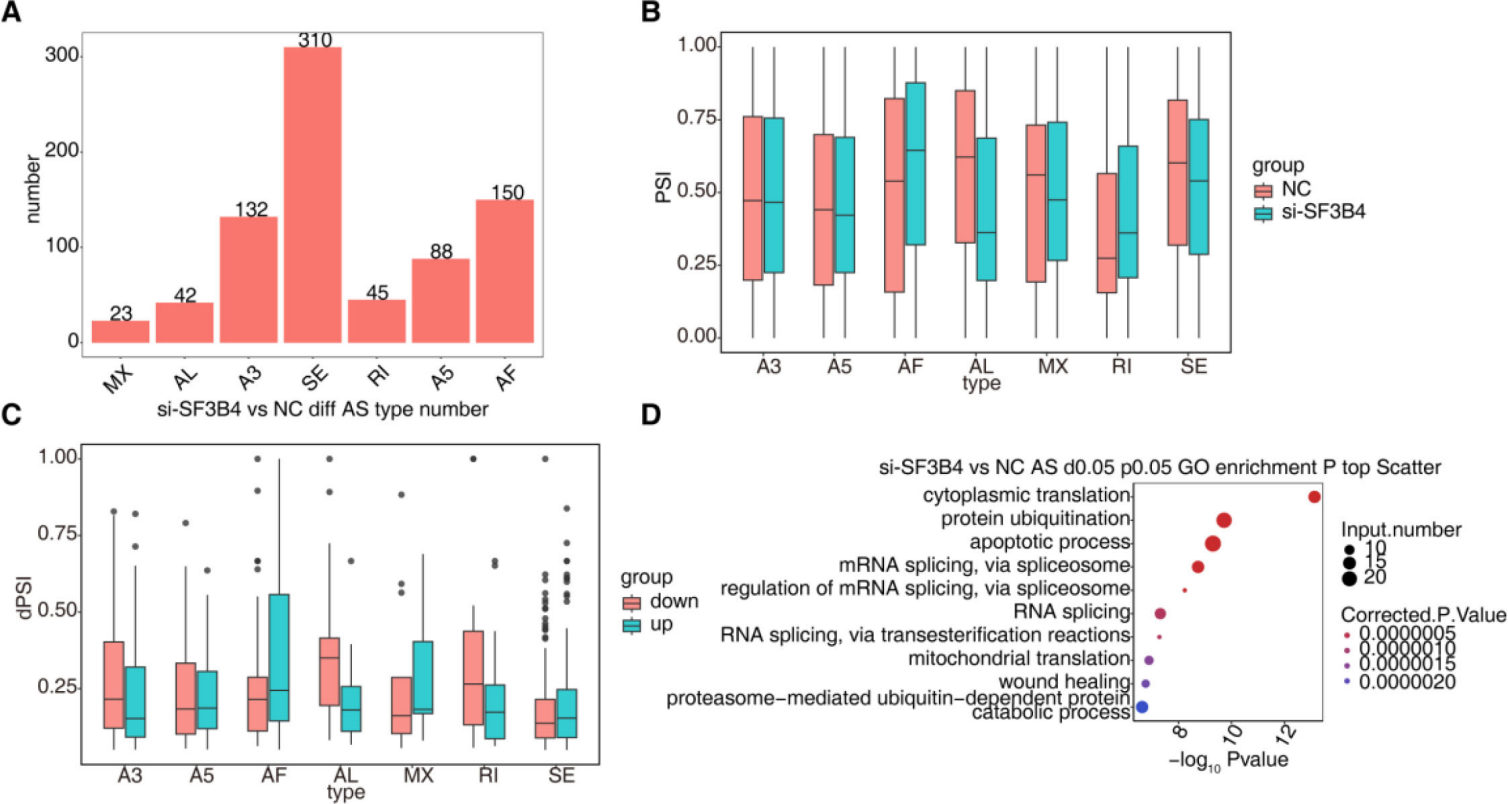
Differential alternative splicing events identified by ONT long-read RNA sequencing after SF3B4 knockdown. **(A)** Numbers of differential AS events between si-SF3B4 and NC groups, classified by AS type. **(B)** Distribution of percent spliced in (PSI) values for differential AS events in si-SF3B4 and NC groups. **(C)** Distribution of ΔPSI (dPSI) values for differential AS events between si-SF3B4 and NC groups. **(D)** GO biological process enrichment analysis of genes associated with differential AS events.

By quantifying different types of AS events (**Figure 5A**), approximately 790 differential AS events (P < 0.05) were identified, predominantly comprising exon skipping (SE, 310), alternative 3′ splice site usage (A3, 132), alternative 5′ splice site usage (A5, 150), intron retention (RI, 88), mutually exclusive exons (MX, 23), alternative first exon (AF, 45), and alternative last exon (AL, 42). This indicates that SF3B4 knockdown induces widespread AS reprogramming. Further analysis of the Percent Spliced In (PSI) values for different AS types in the SF3B4 knockdown and control groups (**Figure 5B**) revealed that SF3B4 knockdown significantly altered the usage patterns of splicing events across various AS types. Examination of the ΔPSI (dPSI) values for differential AS events (**Figure 5C**) showed that SF3B4 knockdown caused both upregulation and downregulation of several SE, A5, and AF events, highlighting SF3B4’s bidirectional role in regulating exon-selective splicing. To explore the functional significance of these differential AS events, GO functional enrichment analysis was performed on the associated genes (**Figure 5D**). The results showed significant enrichment in pathways such as mRNA splicing, RNA processing, protein ubiquitination, apoptosis, mitochondrial translation, and protein degradation, suggesting that SF3B4 regulates critical AS events in HCC-related genes, thereby contributing to tumor cell proliferation, metabolism, and survival.

### 3.6 SF3B4 binds to the 3’UTR region of SREBF1, thereby enhancing its stability

**Figure 6:**
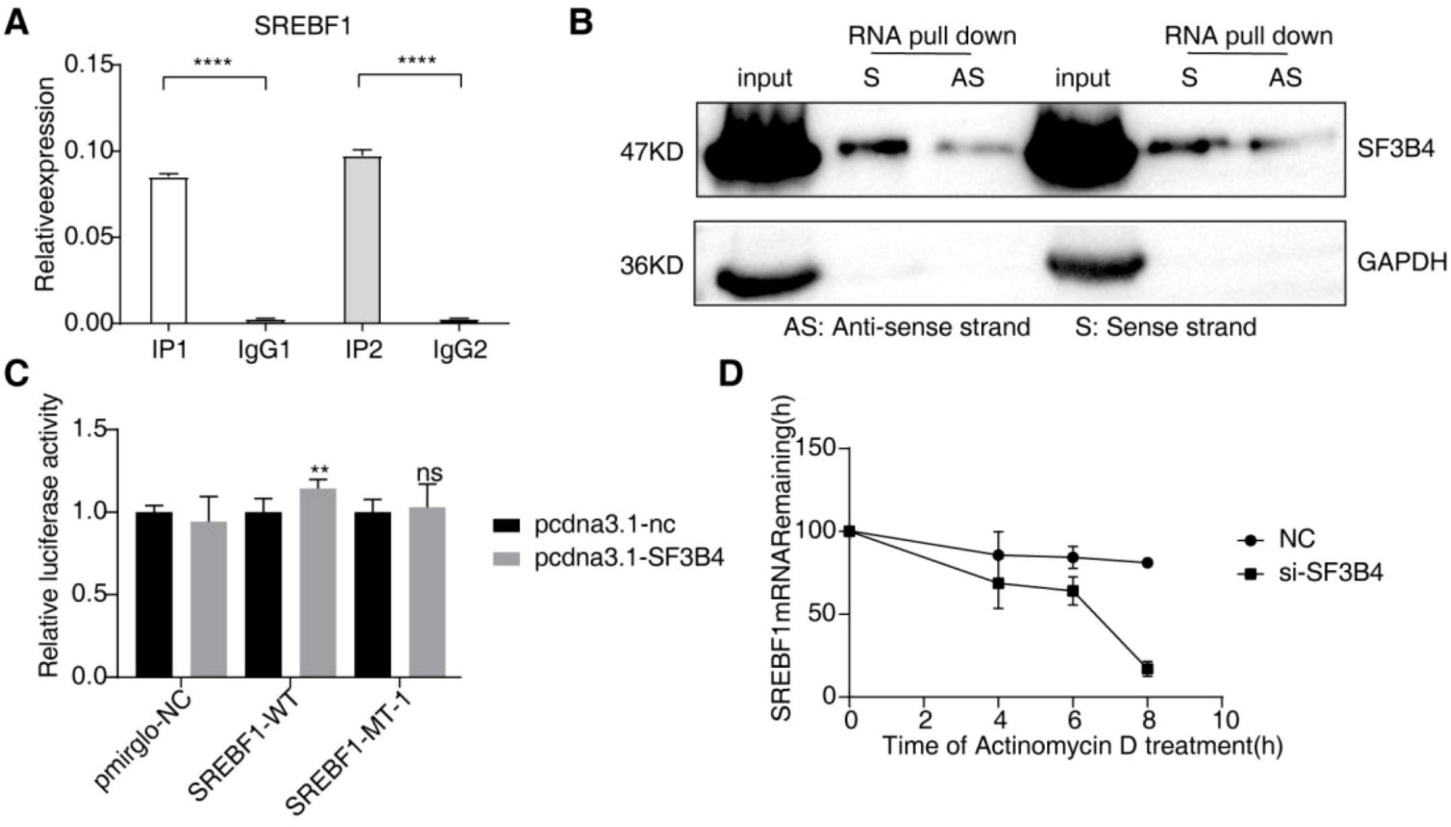
SF3B4 binds to the 3′UTR of SREBF1 and enhances its mRNA stability. **(A)** RIP–qPCR analysis validating the binding of SF3B4 to the 3′UTR of the SREBF1 transcript. **(B)** RNA pull-down confirming the interaction between SREBF1 RNA and SF3B4 protein. **(C)** Dual-luciferase reporter assay showing that SF3B4 binds to the SREBF1 3′UTR and enhances reporter activity, whereas mutation of the binding site abolishes this effect. **(D)** Actinomycin D chase assay demonstrating that SF3B4 knockdown reduces SREBF1 mRNA stability. ns: no significance; ***p<0.001; ****p<0.0001.

To further confirm the direct binding of SF3B4 to SREBF1 and its regulatory effect on mRNA stability, RIP, RNA pull-down, dual-luciferase reporter, and mRNA stability assays were conducted. The RIP results showed that after immunoprecipitating SF3B4, SREBF1 mRNA was significantly enriched in the IP1 and IP2 groups, while no detection was observed in the IgG control group (P < 0.0001), indicating a direct binding interaction between SREBF1 mRNA and SF3B4 (**Figure 6A**). The RNA pull-down experiment further validated this interaction, as the sense strand (S) probe of SREBF1 effectively captured the SF3B4 protein, while the antisense strand (AS) showed no binding signal, confirming that SF3B4 specifically recognizes and binds to the SREBF1 transcript (**Figure 6B**). To examine the role of SF3B4 in post-transcriptional regulation of SREBF1, a dual-luciferase reporter assay was performed. The results showed that overexpression of SF3B4 significantly increased the fluorescence activity of the wild-type SREBF1 3′UTR reporter, while mutation of the binding site weakened this effect significantly (P < 0.01), suggesting that SF3B4 stabilizes SREBF1 by binding to specific sites in its 3′UTR (**Figure 6C**). Lastly, Actinomycin D was used to inhibit transcription, and the decay of SREBF1 mRNA was measured at various time points. The results indicated that SF3B4 knockdown significantly accelerated the degradation of SREBF1 mRNA, shortening its half-life compared to the control group (**Figure 6D**).

### 3.7 The binding of SF3B4 to SREBF1 promotes the invasion and migration of HCC

**Figure 7:**
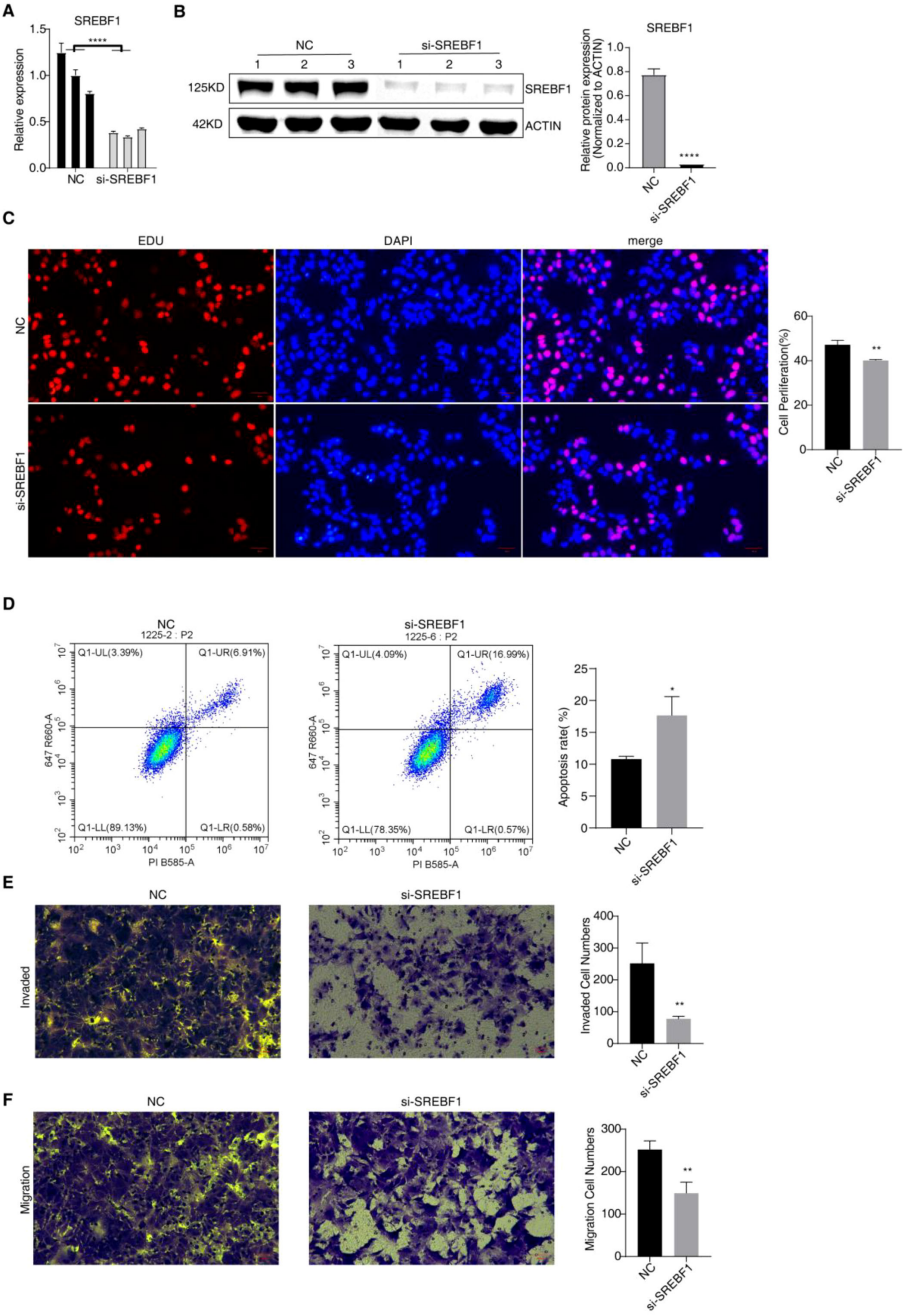
Effects of SREBF1 knockdown on the malignant phenotypes of Huh-7 cells. **(A)** qRT–PCR analysis confirming the knockdown efficiency of SREBF1 in Huh-7 cells. **(B)** Western blot analysis verifying the reduction of SREBF1 protein expression following si-SREBF1 transfection. **(C)** EdU incorporation assay showing reduced proliferation of Huh-7 cells after SREBF1 knockdown. **(D)** Flow cytometric analysis of apoptosis in Huh-7 cells following SREBF1 silencing. **(E)** Transwell invasion assay demonstrating decreased invasive capacity of Huh-7 cells after SREBF1 knockdown. **(F)** Transwell migration assay showing reduced migratory ability of Huh-7 cells upon SREBF1 silencing. * *p*<0.05; **p<0.01; ****p<0.0001.

**Figure 8:**
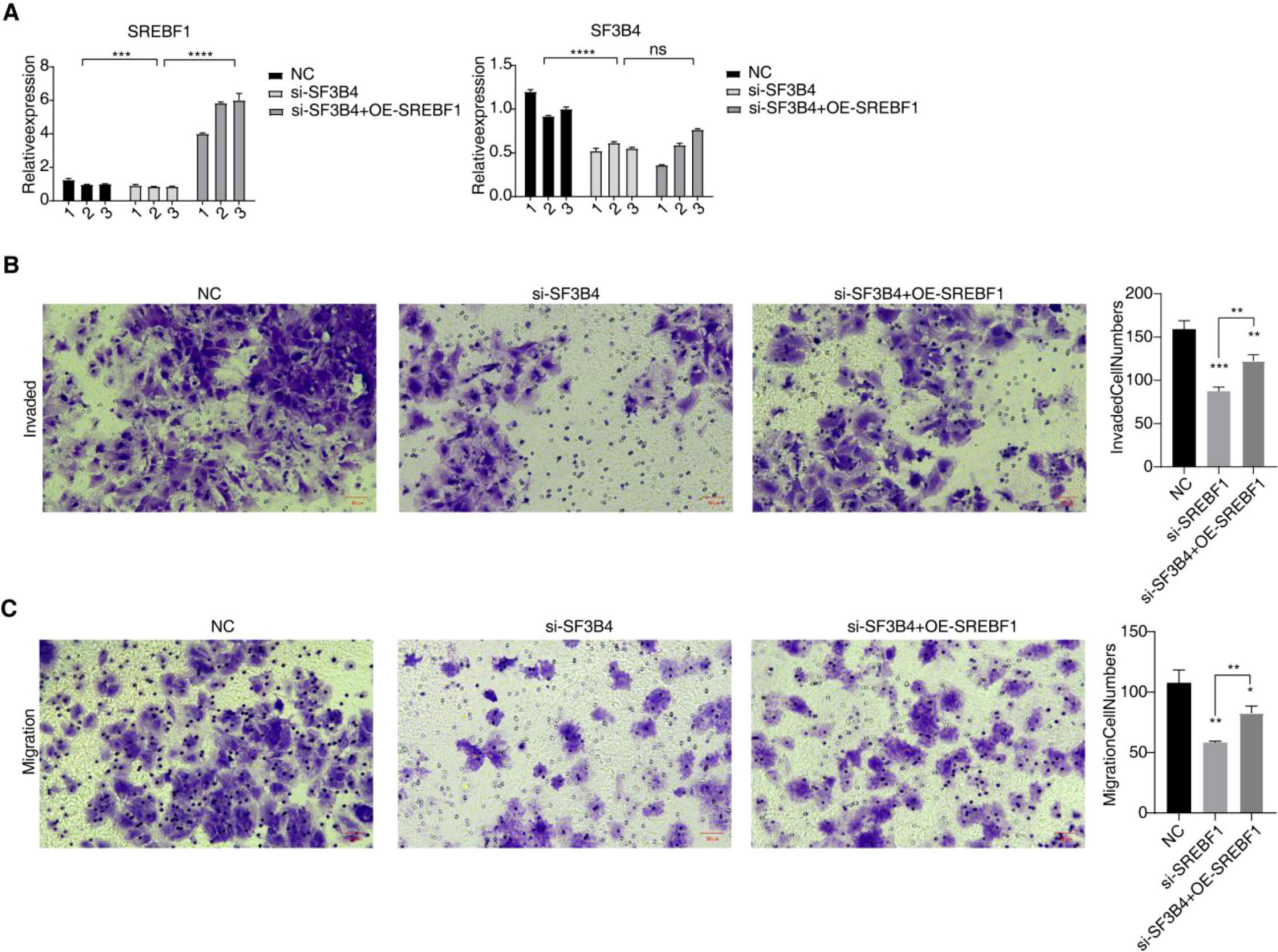
SREBF1 overexpression functionally reverses the suppressive effects of SF3B4 silencing in HCC cells. **(A)** qRT–PCR analysis confirming efficient SF3B4 knockdown and concomitant SREBF1 overexpression in Huh-7 cells. **(B)** Transwell invasion assay showing that enforced SREBF1 expression partially restores the invasive capacity of Huh-7 cells impaired by SF3B4 silencing. **(C)** Transwell migration assay demonstrating that SREBF1 overexpression functionally reverses the reduced migratory ability induced by SF3B4 knockdown. ns: no significance; * *p*<0.05; **p<0.01; ***p<0.001; ****p<0.0001.

**Figure 9:**
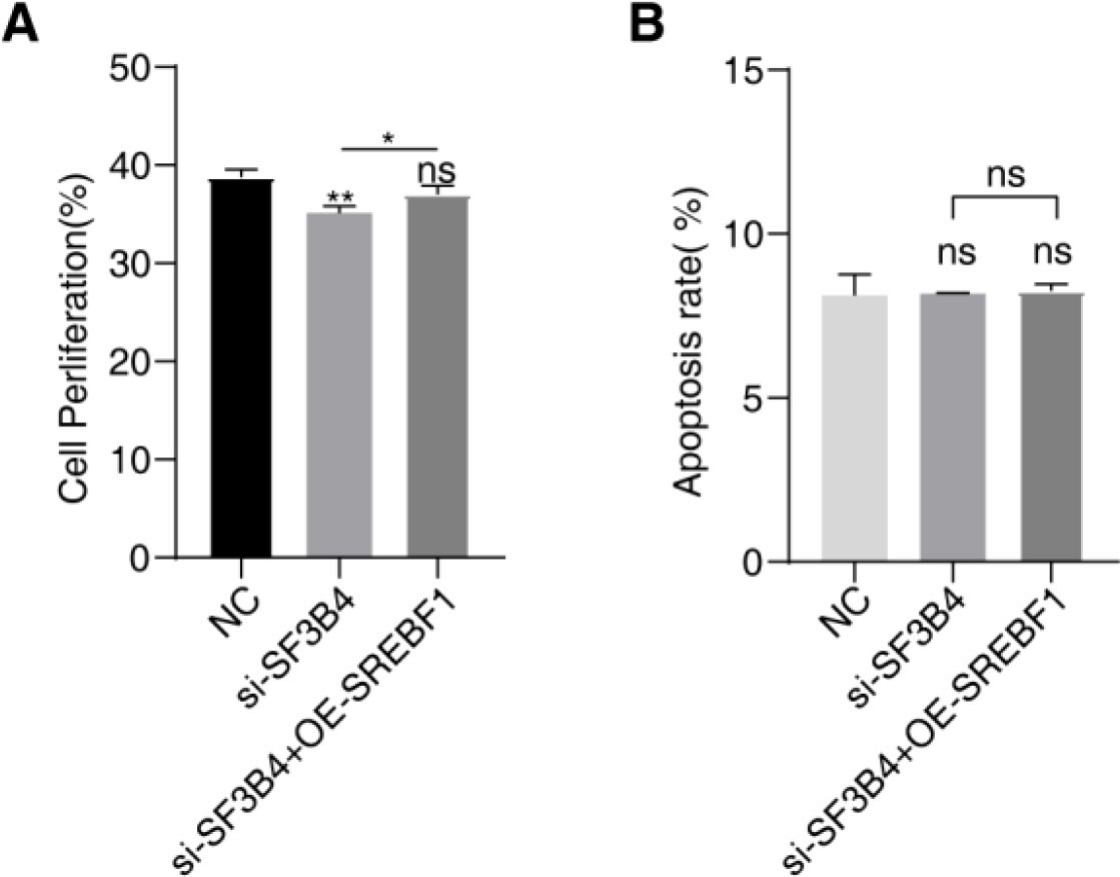
Effects of SF3B4 knockdown and SREBF1 overexpression on proliferation and apoptosis of Huh-7 cells. **(A)** Cell proliferation analysis showing that SREBF1 overexpression partially reverses the reduced proliferative capacity of Huh-7 cells induced by SF3B4 knockdown. **(B)** Apoptosis analysis demonstrating no significant change in apoptotic rate following SF3B4 silencing or SREBF1 overexpression. ns: no significance; * *p*<0.05; **p<0.01.

qRT–PCR and Western blot assays confirmed that in the siRNA-mediated SREBF1 knockdown group, both mRNA and protein levels of SREBF1 were significantly reduced, demonstrating effective knockdown efficiency (**Figures 3**-**9A, B**). The EdU assay showed that SREBF1 knockdown markedly inhibited the proliferation of Huh-7 cells (**Figures 3**-**9C**, P < 0.01). Flow cytometric analysis revealed that, compared to the control group, silencing SREBF1 significantly increased the apoptosis rate of Huh-7 cells (**Figure 3**-**9D**, *P* < 0.05). In invasion and migration assays, SREBF1 knockdown significantly decreased the number of cells passing through the Matrigel basement membrane and reduced the migratory capacity of Huh-7 cells (**Figures 3**-**9E, F**, *P* < 0.01). These results indicate that SREBF1 promotes cell proliferation, invasion, and migration, while inhibiting apoptosis, suggesting its role in promoting HCC tumorigenesis.

### 3.8 The overexpression of SREBF1 reverses the inhibitory effect of SF3B4 knockdown on the invasion and migration ability of HCC

Further investigation into the impact of SF3B4 binding to SREBF1 in HCC was conducted through rescue experiments. In Huh-7 cells, SF3B4 was knocked down, and SREBF1 was overexpressed in the SF3B4-knockdown cells. qRT–PCR results confirmed successful SF3B4 knockdown and SREBF1 overexpression (**Figure 8A**). SF3B4 knockdown inhibited the invasion and migration of Huh-7 cells, while overexpression of SREBF1 partially reversed this inhibition (**Figure 8B, C**). These results suggest that SF3B4 promotes cell migration and invasion through binding and stabilizing SREBF1 expression, without affecting cell proliferation or apoptosis.

### 3.9 Detection of proliferation and apoptosis of Huh-7 cells after SF3B4 knockdown and SREBF1 overexpression

Knocking down SF3B4 inhibited the proliferation of Huh-7 cells, and this proliferation defect was partially restored upon SREBF1 overexpression (**Figure 9A**). However, no significant changes in apoptosis were observed following SF3B4 silencing and SREBF1 overexpression (**Figure 9B**).

### 3.10 Knockdown of SREBF1 leads to significant changes in the transcriptome remodeling and related biological processes of HCC

**Figure 10:**
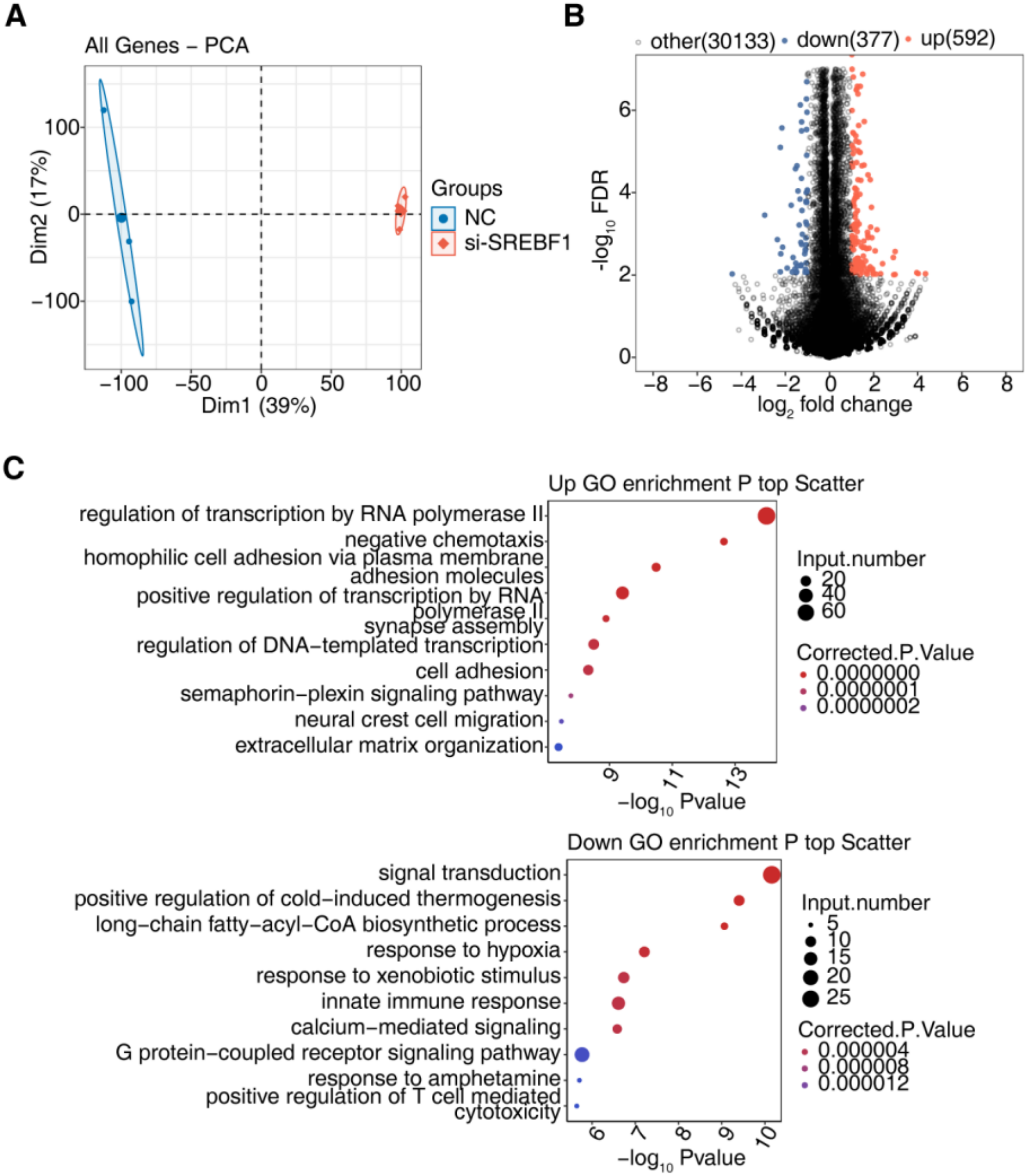
Transcriptomic profiling of Huh-7 cells following SREBF1 knockdown. **(A)** PCA based on the expression profiles of all genes, showing clear separation between NC and si-SREBF1 groups. **(B)** Volcano plot displaying differentially expressed genes (DEGs) identified after SREBF1 knockdown. **(C)** GO biological process enrichment analysis of upregulated (UP) and downregulated (DOWN) DEGs.

Transcriptome sequencing analysis was performed on HCC cells with SREBF1 knockdown. Principal component analysis (PCA) was initially used to visualize and reduce the dimensionality of the overall gene expression profiles. The results revealed distinct separation between the si-SREBF1 and NC control groups along principal components 1 (Dim1) and 2 (Dim2) (**Figure 10A**), indicating that SREBF1 knockdown significantly altered the global transcriptional landscape of HCC cells. Differential expression analysis identified 969 DEGs, with 592 upregulated and 377 downregulated genes (**Figure 10B**). To explore the potential biological functions of these DEGs, GO enrichment analysis was conducted separately for the upregulated and downregulated genes. The upregulated genes were primarily enriched in pathways related to “RNA polymerase II-mediated transcriptional regulation,” “homophilic cell adhesion,” “positive transcriptional regulation,” and “DNA template-dependent transcription” (**Figure 10C**), suggesting that SREBF1 knockdown may activate processes related to gene expression and cell adhesion. Conversely, downregulated genes were significantly enriched in pathways related to “signal transduction,” “long-chain fatty acid metabolism,” “immune response,” “G protein-coupled receptor signaling pathway,” and “cytotoxicity mediated by T cells,” indicating that SREBF1 may play a critical role in regulating metabolic reprogramming, immune regulation, and cell signaling in HCC.

### 3.11 SF3B4 and SREBF1 synergistically regulate the transcriptome expression profile of HCC

**Figure 11:**
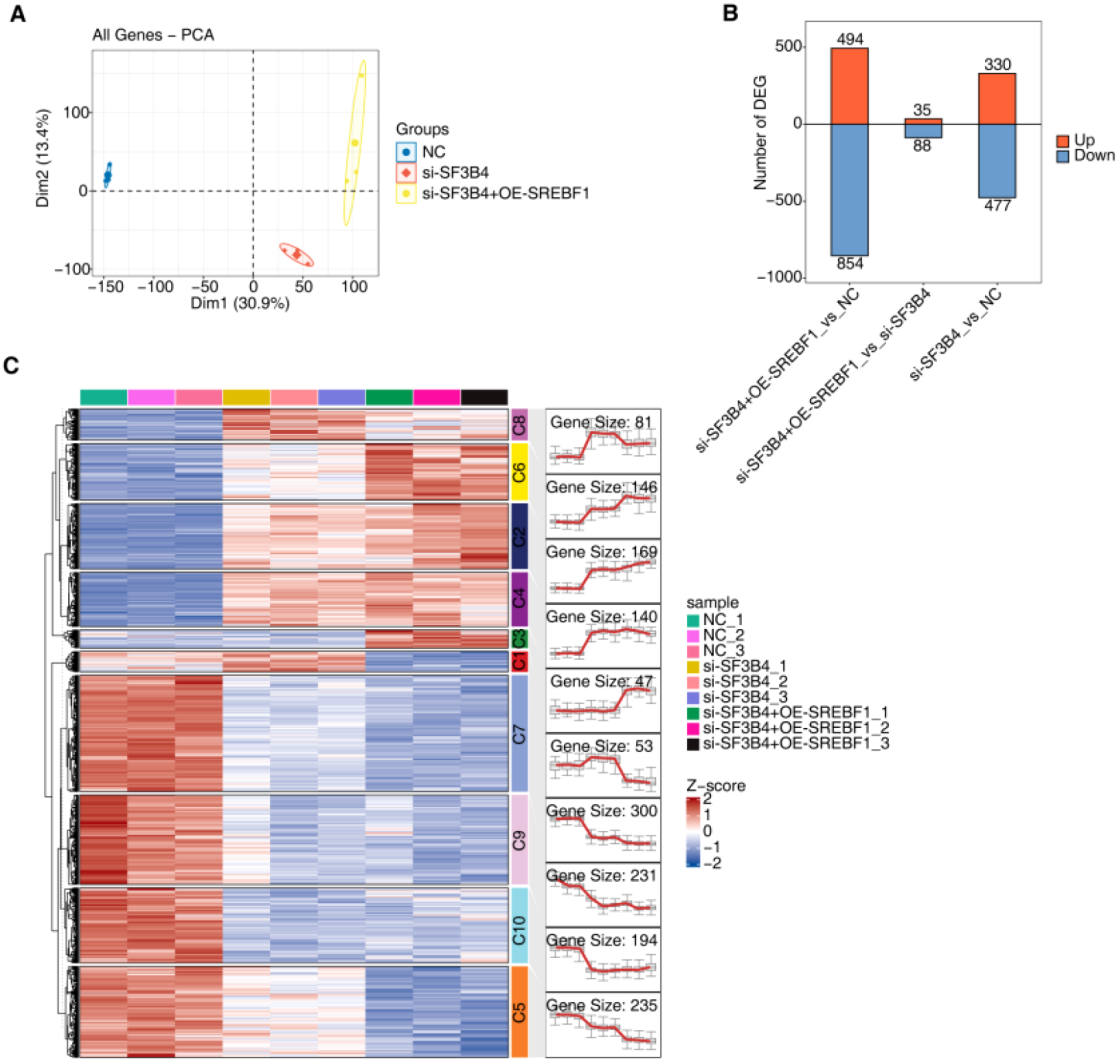
RNA-seq analysis of Huh-7 cells with SF3B4 knockdown and SREBF1 overexpression. **(A)** PCA based on the expression profiles of all genes in NC, si-SF3B4, and si-SF3B4 + OE-SREBF1 groups. **(B)** Bar plot showing the numbers of upregulated (UP) and downregulated (DOWN) differentially expressed genes among the three groups. **(C)** Clustered heatmap of differentially expressed genes generated by clusterGVis, illustrating distinct transcriptional patterns across NC, si-SF3B4, and si-SF3B4 + OE-SREBF1 samples.

Transcriptome sequencing was conducted to investigate the effects of SF3B4 silencing and SREBF1 overexpression on gene expression in liver cancer cells. PCA revealed clear separation between the NC, si-SF3B4, and si-SF3B4 + OE-SREBF1 groups at the transcriptional level, indicating that SF3B4 silencing and SREBF1 overexpression substantially altered gene expression patterns (**Figure 11A**). DEG analysis identified 894 DEGs in the si-SF3B4 group compared to the NC group, including 494 upregulated and 400 downregulated genes. In contrast, 365 DEGs were detected between the si-SF3B4 + OE-SREBF1 and si-SF3B4 groups, with 330 upregulated and 35 downregulated genes, suggesting that SREBF1 overexpression partially reversed the gene expression changes induced by SF3B4 silencing (**Figure 11B**). Heatmap analysis demonstrated a consistent clustering pattern among the three groups, with distinct gene grouping characteristics in each, further supporting the synergistic role of SF3B4 and SREBF1 in the transcriptional regulation of liver cancer cells (**Figure 11C**).

### 3.12 SF3B4 binds to SREBF1 to regulate the expression of downstream genes

**Figure 12:**
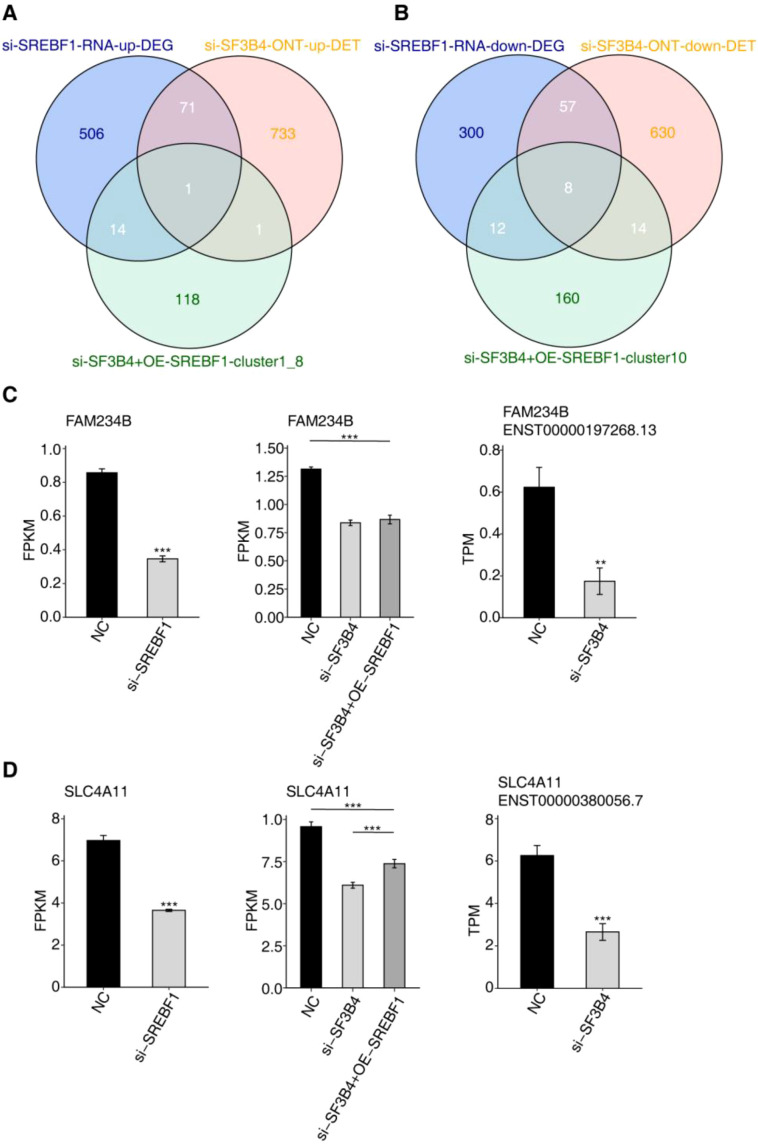
Integrative overlap analysis of si-SF3B4 ONT-lrRNA-seq, si-SREBF1 RNA-seq, and si-SF3B4 + OE-SREBF1 RNA-seq datasets. **(A)** Venn diagram showing the overlap among upregulated genes identified in si-SREBF1 RNA-seq, cluster 1 and cluster 8 genes from si-SF3B4 + OE-SREBF1 RNA-seq, and upregulated differentially expressed transcripts (DETs) identified by si-SF3B4 ONT long-read RNA sequencing. **(B)** Venn diagram illustrating the overlap among downregulated genes from si-SREBF1 RNA-seq, cluster 10 genes from si-SF3B4 + OE-SREBF1 RNA-seq, and downregulated DETs identified by si-SF3B4 ONT long-read RNA sequencing. **(C)** Bar plots showing the expression levels of FAM234B in RNA-seq datasets and the differential expression of corresponding transcripts identified by ONT long-read RNA sequencing. **(D)** Bar plots showing the expression levels of SLC4A11 in RNA-seq datasets and the differential expression of corresponding transcripts identified by ONT long-read RNA sequencing. * *p*<0.05; **p<0.01; ***p<0.001.

By integrating genes from the C1 and C8 groups with significantly upregulated genes from the SREBF1 knockdown RNA-seq data, along with genes from the SF3B4 knockdown ONT-lrRNA-seq data and upregulated transcripts, one gene was identified that exhibited increased expression following both SF3B4 and SREBF1 knockdown, but decreased expression upon SREBF1 overexpression. Additionally, a significant upregulated differential transcript was observed after SF3B4 knockdown (**Figure 12A**). Further integration of genes from the C10 group with significantly downregulated genes from the SREBF1 knockdown RNA-seq data and genes from the SF3B4 knockdown ONT-lrRNA-seq data with downregulated transcripts revealed eight genes that showed decreased expression after both SF3B4 and SREBF1 knockdown, with increased expression upon SREBF1 overexpression, alongside a notable downregulated differential transcript following SF3B4 knockdown (**Figure 12B**). Notably, FAM234B and SLC4A11, both associated with cancer progression, displayed differential gene and transcript expression levels as shown in (**Figures 12C, D**).

### 3.13 *In vivo* tumorigenesis experiments of SF3B4 and SREBF1

**Figure 13:**
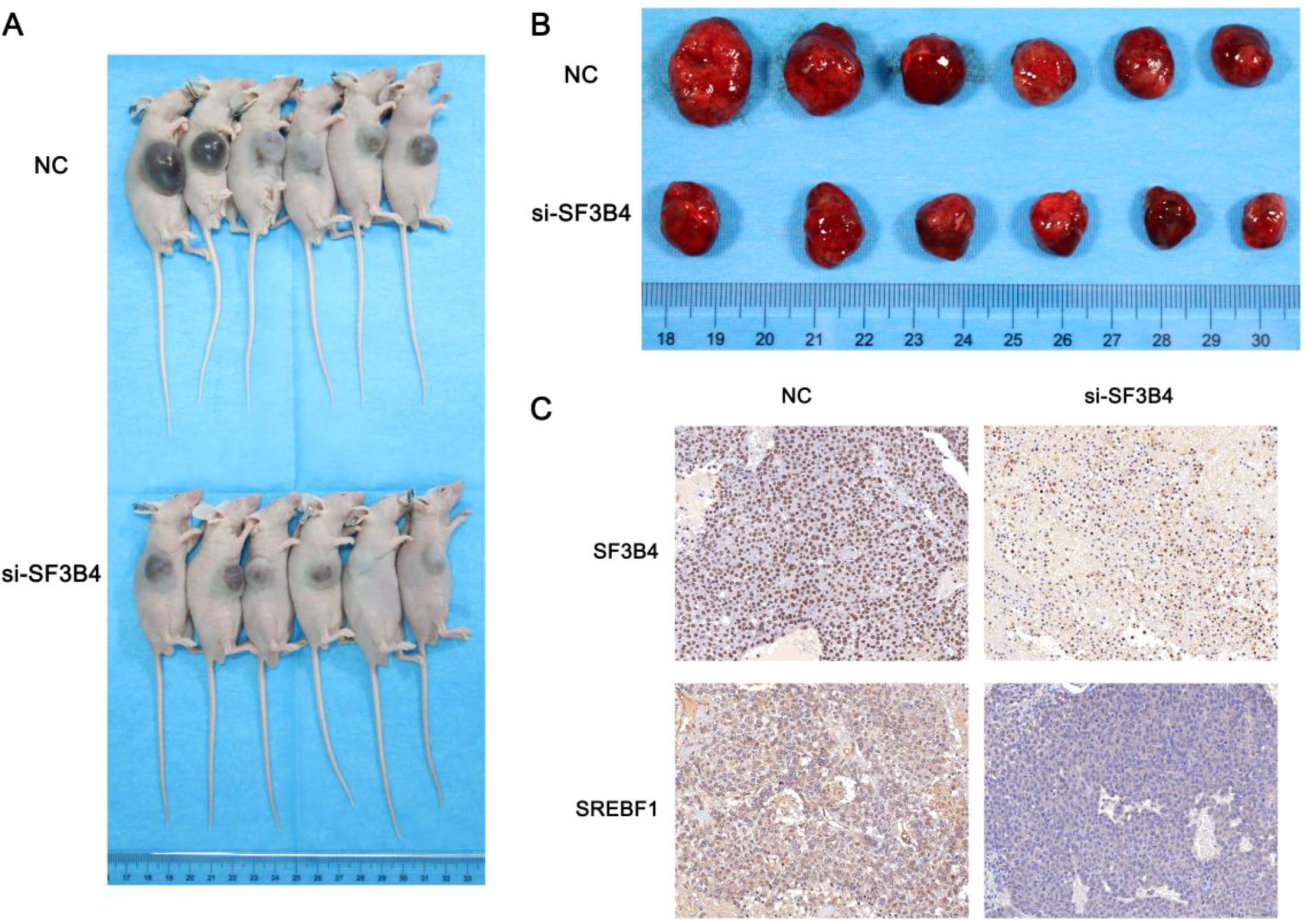
SF3B4 knockdown suppresses tumor growth *in vivo* and reduces SREBF1 expression. **(A)** SF3B4 silencing inhibits xenograft tumor growth. Representative images of nude mice bearing subcutaneous xenografts derived from NC or si-SF3B4–transfected Huh-7 cells. **(B)** Reduced tumor burden upon SF3B4 knockdown. Gross morphology of excised tumors from NC and si-SF3B4 groups, showing decreased tumor size in the si-SF3B4 group. **(C)** Decreased SF3B4 and SREBF1 expression in si-SF3B4 tumors. Immunohistochemical staining of SF3B4 and SREBF1 in xenograft tumor tissues from NC and si-SF3B4 groups.

Subcutaneous xenograft experiments demonstrated that SF3B4 knockdown significantly inhibited tumor growth *in vivo*. As shown in **Figures 13A, B**, tumors derived from si-SF3B4-treated cells were notably smaller than those in the NC group. Immunohistochemical analysis further confirmed a reduction in SF3B4 expression in the si-SF3B4 tumors, accompanied by a marked decrease in SREBF1 expression (**Figure 13C**). These results suggest that SF3B4 promotes HCC tumor growth *in vivo*, at least partially by maintaining SREBF1 expression.

### 3.14 Clinical sample validation of SF3B4 and SREBF1

**Figure 14:**
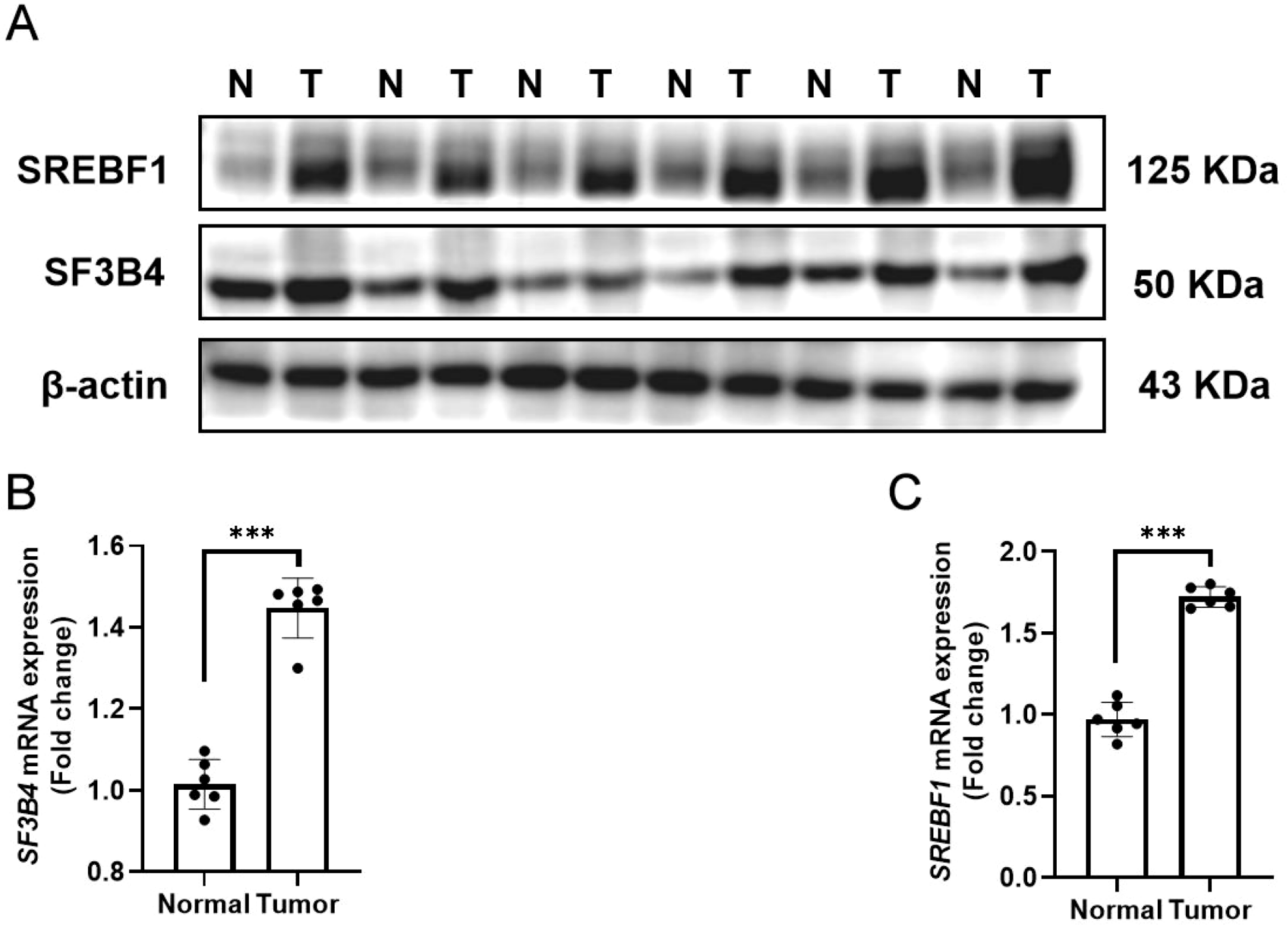
Clinical validation of SF3B4 and SREBF1 expression in hepatocellular carcinoma. **(A)** Western blot analysis of SF3B4 and SREBF1 protein expression in tumor tissues (T) and paired adjacent normal tissues (N) from six patients with HCC. **(B)** RT–qPCR analysis of SF3B4 mRNA expression in the same paired HCC tissues. **(C)** RT–qPCR analysis of SREBF1 mRNA expression in the same paired HCC tissues. ***p<0.001.

**Figure 15:**
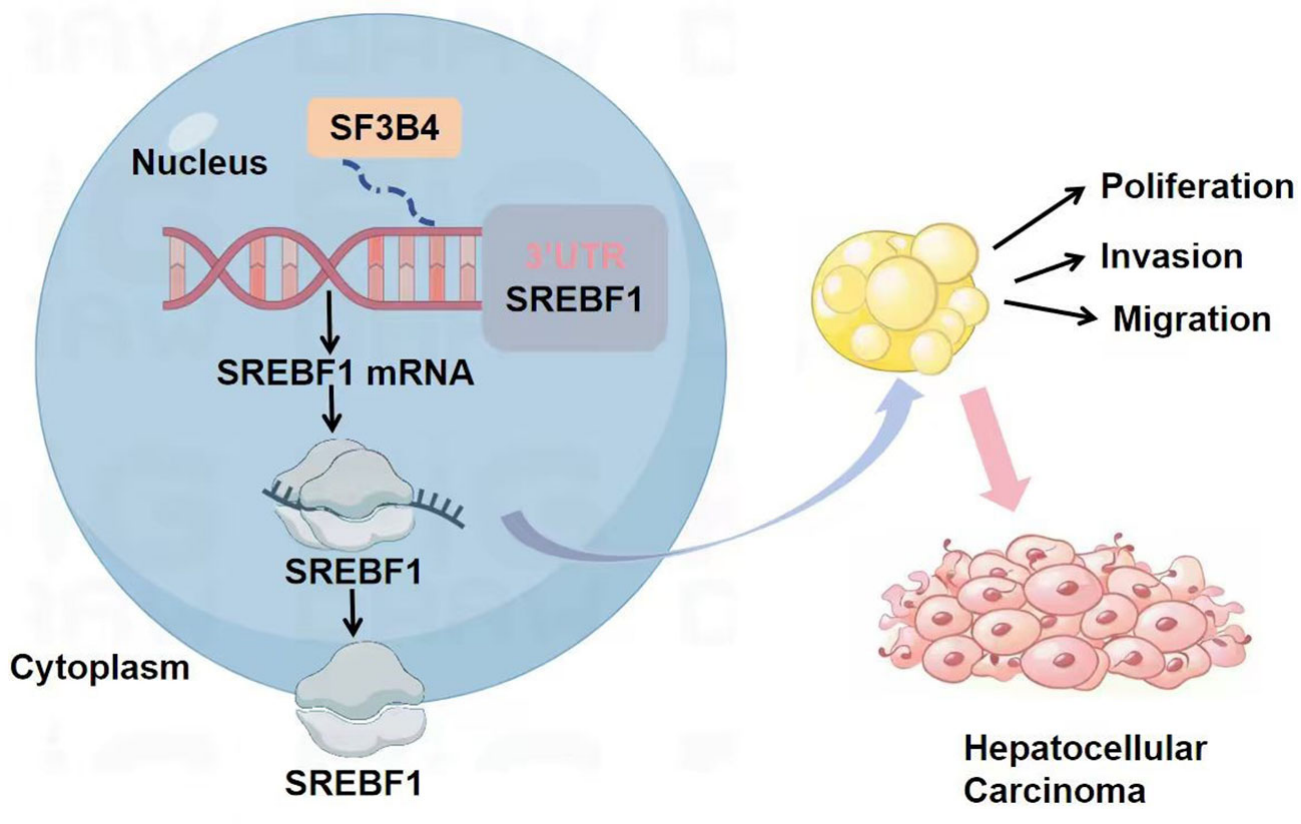
Schematic representation of SF3B4 and SREBF1 function and mechanisms of action in HCC.

To investigate the expression levels of SF3B4 and SREBF1 in HCC and their potential correlation, protein and mRNA analyses were conducted on paired clinical samples (tumor tissue, T, and corresponding normal tissue, N). As shown in **Figure 14A**, Western blot analysis revealed significantly higher protein levels of both SREBF1 and SF3B4 in tumor tissues compared to normal tissues. Furthermore, RT-qPCR analysis confirmed that the transcriptional levels of SF3B4 and SREBF1 were also significantly elevated in liver cancer tissues (**Figures 14B, C**), with the differences being statistically significant (***P < 0.001). This result aligns with the expression pattern observed in the *in vitro* cell experiments, further supporting the co-elevated expression of SF3B4 and SREBF1 in HCC. Schematic diagram illustrating the functions and mechanisms of SF3B4 and SREBF1 in HCC (**Figure 15**).

## 4 Discussion

HCC is a major contributor to the global cancer burden, ranking as the third leading cause of cancer-related deaths worldwide (19). Representing 75-85% of all primary liver cancers, HCC is most often diagnosed at advanced stages, which contributes to its unfavorable prognosis (20). The global distribution of HCC is highly heterogeneous, largely due to regional differences in etiological risk factors (21). Approximately 72% of HCC cases occur in Asia, with China alone accounting for more than half of the global disease burden (22). Europe, Africa, North America, and Latin America contribute roughly 10%, 7.8%, 5.1%, and 4.6% of cases, respectively (23).While age-standardized incidence rates of HCC have plateaued or declined in certain populations, including those in China, Japan, and the United States, the overall number of HCC cases continues to rise globally, driven by population growth and aging (24). Recent evidence suggests that multi-biomarker models outperform single markers in HCC detection. Notably, the ASAP score demonstrates superior diagnostic performance compared with the GALAD model across different etiologies of chronic liver disease, including early-stage HCC (25).

Aberrant RNA processing, including AS and transcript stability regulation, has emerged as a hallmark of cancer (26). Splicing factors and RBPs are increasingly recognized for their multifunctional roles that extend beyond canonical spliceosome assembly, influencing RNA turnover, localization, and translation (27). Consistent with this, our transcriptome-wide analyses revealed that SF3B4 knockdown induces extensive transcriptional and splicing reprogramming, affecting genes involved in apoptosis, metabolism, immune response, and signal transduction. These findings highlight the central role of SF3B4 in shaping the malignant transcriptome of HCC cells.

Previous studies have demonstrated that SF3B4 promotes tumor progression through AS regulation in various cancers (28). In HCC, our earlier work highlighted SF3B4’s regulation of proliferation and apoptosis through splicing-associated interactions with oncogenic factors (17). However, whether SF3B4 exerts oncogenic functions via splicing-independent, post-transcriptional mechanisms was previously unclear. This study expands the functional scope of SF3B4 by identifying its role in stabilizing SREBF1 mRNA, uncovering a non-canonical mechanism through which SF3B4 drives HCC progression.

Metabolic reprogramming, particularly lipid metabolism, is a defining feature of HCC due to the liver’s central role in lipid synthesis and energy homeostasis (29). SREBF1 is a master transcriptional regulator of *de novo* lipogenesis and has been widely implicated in HCC growth, metastasis, and poor prognosis (30). In Squamous cell carcinomas, the interaction and synergy between SREBF1 and the master transcription factors regulate lipid metabolism and tumor-promoting pathways (31).While transcriptional and signaling-based regulation of SREBF1 has been well studied, its post-transcriptional regulation in liver cancer remains poorly understood. Our data provide evidence that SF3B4 binds to the 3′UTR of SREBF1 mRNA, prolongs its half-life, and maintains its expression. This discovery adds a new layer of regulation to SREBF1 control and highlights the importance of RNA stability in regulating metabolic oncogenes.

Functionally, this study demonstrates that SREBF1 knockdown recapitulates the phenotypic effects of SF3B4 silencing, including suppressed proliferation, invasion, and migration, alongside increased apoptosis. Geng et al. had demonstrated that SREBF1could regulate lipid synthesis and lipid phagocytosis, maintaining lipid homeostasis and tumor growth (32). Overexpression of SREBF1 partially rescues the inhibitory effects of SF3B4 knockdown on malignant behaviors and global transcriptional programs. These rescue experiments provide strong causal evidence that SREBF1 is a key downstream effector of SF3B4 in HCC. The incomplete rescue suggests that SF3B4 regulates additional oncogenic targets, consistent with its broad RNA-binding activity.

Notably, the biological relevance of the SF3B4–SREBF1 axis was confirmed both *in vivo* and in clinical samples. SF3B4 knockdown significantly suppressed xenograft tumor growth and reduced SREBF1 expression in tumor tissues, reinforcing the physiological significance of this pathway. Additionally, paired clinical samples showed concurrent upregulation of SF3B4 and SREBF1 at both mRNA and protein levels in HCC tissues, highlighting the clinical relevance of this regulatory axis.

From a translational perspective, our findings carry several critical implications. First, SF3B4 represents a promising therapeutic target that links RNA metabolism dysregulation with metabolic reprogramming, two key hallmarks of HCC. Targeting SF3B4 may simultaneously disrupt aberrant splicing programs and destabilize metabolic oncogenes like SREBF1. Second, with the growing interest in RNA-based therapies and metabolic interventions for liver cancer, the SF3B4–SREBF1 axis presents a novel avenue for therapeutic development. Additionally, SF3B4-regulated transcripts could serve as biomarkers for patient stratification or predictors of metabolic vulnerability in HCC.

This study has several limitations. While direct binding was identified between SF3B4 and the SREBF1 3′UTR, the precise structural determinants and potential interactions with other RBPs remain unclear. Furthermore, the impact of SF3B4-mediated metabolic regulation on the tumor immune microenvironment warrants further exploration. Future studies using patient-derived xenografts and genetically engineered mouse models will be critical to fully assess the therapeutic potential of targeting this pathway.

In summary, our study uncovers a novel post-transcriptional mechanism through which SF3B4 stabilizes SREBF1 mRNA via direct binding to its 3′UTR, thereby promoting lipogenic reprogramming and HCC progression. These findings expand the functional scope of splicing factors in liver cancer and offer mechanistic insights with potential clinical and therapeutic applications.

## Conclusion

This study identified a previously unrecognized post-transcriptional mechanism by which the splicing factor SF3B4 promotes HCC progression. Our results demonstrate that SF3B4 is aberrantly upregulated in HCC and functions as an oncogenic RBP, directly binding to the 3′UTR of SREBF1 mRNA, enhancing its stability, and sustaining its expression. Through this mechanism, SF3B4 facilitates lipogenic reprogramming and drives malignant phenotypes, including enhanced proliferation, invasion, migration, and tumor growth *in vivo*. These findings establish the SF3B4–SREBF1 axis as a critical molecular link between RNA metabolism dysregulation and metabolic remodeling in HCC.

## Data Availability

The raw sequence data reported in this paper have been deposited in the Genome Sequence Archive (33) in National Genomics Data Center (34), China National Center for Bioinformation / Beijing Institute of Genomics, Chinese Academy of Sciences (GSA-Human: HRA018431) that are publicly accessible at https://ngdc.cncb.ac.cn/gsa-human.
